# Von der anatomischen Lehrsektion zum Unterricht am Krankenbett – eine geschichtliche Würdigung

**DOI:** 10.1007/s10354-021-00836-8

**Published:** 2021-04-14

**Authors:** Ulrich Koehler, Olaf Hildebrandt, Julian Koehler, Wulf Hildebrandt

**Affiliations:** 1 Klinik für Innere Medizin, SP Pneumologie, Intensiv- und Schlafmedizin, Universitätsklinikum Gießen und Marburg GmbH, Philipps-Universität Marburg, Baldingerstr. 1, 35033 Marburg, Deutschland; 2 Medizinische Zellbiologie, Institut für Anatomie und Zellbiologie, Philipps-Universität Marburg, Marburg, Deutschland

**Keywords:** Anatomie, Sektion, Klinischer Unterricht, Unterricht am Krankenbett, Anatomy, Autopsy, Clinical tuition, Bedside teaching

## Abstract

Zu allen Zeiten waren die Anatomen bemüht, der Medizin wissenschaftliche Grundlagen zu vermitteln. Der Anatom hat den toten Körper zergliedert, um damit den Lebenden zu dienen. Das Verständnis physiologischer und pathophysiologischer Prozesse setzt die Kenntnis der Anatomie voraus. Im Corpus Hippocraticum findet man keinen sicheren Hinweis auf die Durchführung menschlicher Sektionen. In Alexandria wurde um 300 vor Christus zum ersten Mal Anatomie an der menschlichen Leiche gelehrt. Mehr als 1300 Jahre standen die Anatomie und die Heilkunde dann unter dem Einfluss des Galen von Pergamon (131–201 n. Chr.). Der Italiener Mondino dei Luzzi (1275–1326) war der Erste, der den systematischen Anatomieunterricht unter regelmäßiger Einbeziehung von Lehrsektionen in den Lehrbetrieb in Bologna eingeführt hat. Andreas Vesalius (1514–1564) aus Belgien hat in der Neuzeit die wissenschaftlich fundierte Humananatomie begründet und viele Fehler der von Galen tradierten Ansichten der Anatomie korrigiert. Im 17. und 18. Jahrhundert waren die niederländischen Universitäten, allen voran die Universität in Leiden, führend im Hinblick auf die klinische und praktische Studentenausbildung.

## Summary

At all times anatomists endeavored to procure scientific foundations for medicine. The anatomist dissected corpses in order to serve the living. The knowledge of anatomy is a prerequisite for the understanding of physiological and pathophysiological processes. In the “Hippocratic corpus” there is no clear reference to the performance of human autopsies. Anatomy was taught on a human corpse for the first time in Alexandria around 300 B.C. For more than 1300 years anatomy and medicine then stood under the influence of Galen of Pergamon (131–201 A.D.). The Italian Mondino dei Luzzi (1275–1326) was the first to introduce systematic anatomy lessons with a regular inclusion of teaching dissections in the teaching curriculum in Bologna. Andreas Vesalius (1514–1564) from Belgium founded the scientifically based human anatomy during the modern era and corrected many errors in the traditional views on anatomy of Galen. In the seventeenth and eighteenth centuries the Dutch universities, particularly the University of Leiden, were the leaders with respect to the clinical and practical student training.

Ärzte ohne Anatomie sind wie Maulwürfe, sie tappen im Dunkeln und ihrer Hände Arbeit sind Erdhügel. (*Friedrich Tiedemann*, Heidelberger Anatom 1781–1861)

## Anatomie als wissenschaftliches Fundament der klinischen Medizin

Mit dem Tod hat das Leben den Körper verlassen, es bleibt eine Körperhülle (Leiche) zurück. Aristoteles (384–322) vertrat die Auffassung, dass an einer Leiche nichts Menschliches mehr vorhanden sei. Demzufolge sah er es auch nicht als verwerflich an, den Körper eines Menschen zu zergliedern, um sein Inneres zu erkunden. Die Anatomie stellt zweifellos das Fundament medizinischen Wissens dar, sie ist der Schlüssel zum Verständnis der Medizin [1]. In der Geschichte der europäischen Medizin ist die Anatomie das älteste Grundlagenfach.

Anatomie im weitesten Sinne des Wortes ist die Wissenschaft der Organisationen. Sie zerlegt die Organismen in ihre nächsten bildenden Bestandtheile, eruirt das Verhältniss derselben zu einander, untersucht ihre äusseren, sinnlich wahrnehmbaren Eigenschaften und ihre innere Struktur, und sucht aus dem Todten zu lernen, was das Lebendige war. (Joseph Hyrtl; [1])

Da der menschliche Geist sich nicht mit dem gedankenlosen Anschauen der Dinge zufrieden giebt, sondern Plan und Bestimmung auszumitteln sucht, so kann die innige Verbindung der Anatomie mit der Functionenlehre (Physiologie im engeren Sinne) nicht verkannt werden. Die Anatomie ist somit Grundlage der Physiologie, und dadurch zugleich Fundamentalwissenschaft der ganzen Medicin. (Joseph Hyrtl; [1])

Der Begriff Anatomie kommt vom griechischen „anatemnein“ und bedeutet *aufschneiden. Die anatomische Präparation* ist die natürliche Art und Weise, um an Wissen über das Innere des tierischen und menschlichen Körpers zu gelangen. Durch die Sektion war es erstmalig möglich geworden, Medizin auf der Basis von Objektivierbarem aufzubauen. Zwar haben religiöse Verbote und kulturelle Tabus die Durchführung von Sektionen immer wieder eingeschränkt, sie wurden jedoch nicht gänzlich untersagt [2–6]. Im Corpus Hippocraticum findet man keinen Hinweis auf die Durchführung von menschlichen Sektionen. Hippokrates von Kos (460–370 vor Chr.) hat ein geistiges Lehrgebäude geschaffen, die hellenistischen Ärzte haben es um ein pragmatisches erweitert [2, 3, 7, 8]. Aristoteles hat eine Vielzahl an Tieren, aber keinen menschlichen Körper seziert. In Alexandria wurde um 300 vor Christus zum ersten Mal Anatomie an der menschlichen Leiche gelehrt. Besonders die beiden Anatomen Herophilos von Chalkedon (um 300 vor Chr.) und Erasistratos von Julis auf Keos (um 250 vor Chr.) haben sich mit der tierischen und menschlichen Anatomie beschäftigt [2, 8, 9]. Umfangreiche Ergebnisse anatomischen Schrifttums über die Untersuchungen zum Gehirn und zu den Kreislauforganen gingen beim Brand der Bibliothek von Alexandria (47 vor Chr.) verloren.

## 1300 Jahre „Stillstand der Anatomie“ nach Galenos von Pergamon

*Claudius Galenos von Pergamon* (131–201 n. Chr.), genannt Galen, war zweifellos der erfolgreichste Anatom des klassischen Altertums [2, 3, 10, 11]. Als Gladiatorenarzt und kaiserlicher Leibarzt hatte er viele Möglichkeiten, Einblicke in den menschlichen Körper zu erlangen. Galen hat auf die Entwicklung der Medizin über Jahrhunderte hinweg großen Einfluss ausgeübt und ein riesiges bibliografisches Werk hinterlassen („Corpus Galenicum“). Vom Skelett, den Muskeln, dem Nervensystem sowie den inneren Organen hatte Galen vergleichsweise sehr genaue Vorstellungen. Aufgrund der Tatsache, dass er ausschließlich Tierkörper – insbesondere Affen, Schweine und Hunde – seziert und diese Befunde aber eins zu eins auf den Menschen übertragen hat, sind jedoch falsche Vorstellungen hinsichtlich der menschlichen Anatomie entstanden.

Die praktische Anatomie war nach Galen über ein Jahrtausend hinweg nahezu nicht mehr existent. Die medizinischen Gelehrten haben sich damit begnügt die Galen’sche Lehrmeinung, einem Dogma gleich, zu übernehmen und zu lehren. Niemand hat Galens Theorien in Zweifel gezogen. Kritik, so sie denn überhaupt vorgetragen wurde, wurde seitens der Kirche oder der Ärzteschaft zurückgewiesen. Dreizehn Jahrhunderte ist es zu einer Stagnation anatomischen Wissens gekommen! Im Mittelalter gab es keinen medizinischen Fortschritt, Hippokrates und Galen blieben die medizinischen Lehrmeister über Jahrhunderte hinweg [2, 3, 5]. Die klassische Medizin wurde im Mittelalter in der islamischen Welt praktiziert, wo man die Arbeiten Galens übersetzt, fortgeführt und ergänzt hat. Erst mit der „Schule von Salerno“ (ca. 1000–1200) hat es in Europa wieder einen wissenschaftlichen Aufbruch gegeben. Die Ärzte Salernos hatten im 10. Jahrhundert bereits einen hervorragenden Ruf. Ihre ärztlichen Grundsätze waren ausgerichtet nach denen der alexandrinischen Schule. Lehrsektionen und anatomische Studien waren grundsätzlich erlaubt [2, 12, 13]. Da man davon ausging, dass es zwischen der „Anatomia porcis“ und der „Anatomia hominis“ eine große Übereinstimmung gibt, wurden insbesondere Schweine seziert.

## Studentischer Unterricht und Gründung erster Universitäten im Mittelalter

Erst mit der Erfindung der Buchdruckkunst durch Johannes Gutenberg im 15. Jahrhundert war eine neue Form der Vervielfältigung von Wissen geboren. Die Kunst des Lesens und Schreibens war zuvor nicht verbreitet, praktischen Unterricht in Anatomie und am Krankenbett gab es nicht. Das Studium bestand im Auswendiglernen und im Abschreiben, gelehrt wurden Hippokrates, Galen und Avicenna. Die griechische und arabische Sprache war wenig verbreitet, es existierten allenfalls schlechte Übersetzungen aus dem Lateinischen. Ruf und Qualität eines Lehrers bemaßen sich zu damaliger Zeit nach seiner Belesen- und Zitatfähigkeit [4, 14–16]. Es war weitestgehend eine Lehre im Sinne der Erhaltung von Dogmen. Die abendländischen Studenten hat es damalig in Scharen an die italienischen Universitäten gezogen. Vor allen an den Universitäten in Bologna und Padua genoss man eine ungeahnte Freiheit und hatte zudem noch die besten Lehrer [14–17]. Dem Stauferkaiser Friedrich II (1194–1250) hatte Padua nicht nur die Gründung einer Universität (1222), sondern auch die einer Medizinalordnung (1231) zu verdanken [4]. In der Medizinalordnung heißt es wie folgt:

Der Arzt soll besonders die Anatomie des menschlichen Körpers in den Kollegien gelernt haben und in demjenigen Teil der Medizin vollständig ausgebildet sein, ohne den Operationen weder zweckmäßig ausgeführt noch vollkommen ausgeheilt werden können.

Im Jahr der Gründung 1222 wechselte eine Gruppe von Studenten mit ihren Professoren wegen eines Streites mit der Stadt Bologna nach Padua [14]. Dies war problemlos möglich, war doch zu damaliger Zeit die „universitas“ eine weitgehend unabhängige Gemeinschaft mit dem Zweck des Lernens und Lehrens. Man suchte sich den Ort des Studiums nach Kriterien wie Qualität der Professoren sowie Kosten von Miete und Lehre aus. Die Studenten in Padua waren schon 1228 in 4 „nationes“ aufgeteilt, die einen gemeinsamen Rektor gewählt haben. Zur Durchführung des studentischen Unterrichts stand den Studenten noch kein eigenständiges Gebäude zur Verfügung [14]. Die Scholaren bezahlten dem Professor eine bestimmte Summe als Honorar für seine Vorlesungen und als Miete für den zur Verfügung gestellten Unterrichtsraum. In der Regel hat der Professor den Unterricht in seiner eigenen Wohnung abgehalten. Nur im Falle sehr vieler Zuhörer wurden die Vorlesungen in einen Saal der Gemeinde oder Räume des Klosters verlegt. Der Professor war dafür verantwortlich, dass zu Beginn des Studienjahres alle notwendigen Reparaturen der Unterrichtsräumlichkeit ausgeführt waren. War dem nicht so, so mussten die Professoren eine Strafe zahlen.

## Erste Lehrsektionen und anatomischer Unterricht in Bologna und Padua

Das klassische Sektionsszenario zu damaliger Zeit war nach Galen ausgerichtet [2, 6, 14–16]. Nicht etwa Erkenntnisgewinn und eine eigene differenzierte Betrachtung standen im Vordergrund, sondern die Bestätigung des Bekannten. Der Hochschullehrer zitierte Galen, der Prosektor zergliederte nach dessen Anweisung, und die Studenten vernahmen das gesprochene Wort des Demonstrators. Eine Vielzahl anatomischer Irrtümer wurde somit konsequent fortgeführt.

### Bologna

Lehrsektionen wurden in Italien erstmals zu Beginn des 14. Jahrhunderts durchgeführt. Der italienische Anatom und Professor der Medizin Mondino dei Luzzi (1275–1326) war in Bologna tätig [2, 16]. Er stammte aus einer florentinischen Patrizierfamilie, studierte Medizin an der Universität Bologna, wurde um 1300 promoviert und lehrte dort bis zu seinem Tod an der medizinischen Fakultät. Mondino war der Erste, der den systematischen Anatomieunterricht unter regelmäßiger Einbeziehung von Lehrsektionen (Demonstrationen am geöffneten Leichnam) in den Lehrbetrieb einführte [16]. Er galt als der wichtigste Anatomielehrer seiner Zeit. In Bologna war man zu damaliger Zeit hauptsächlich auf die Rechtswissenschaften festgelegt. Der Wunsch, einen Giftmord aufzuklären, führte im Jahr 1302 zu der ersten gerichtsärztlichen Leichenöffnung. Mondino veröffentlichte 1316 eine Sammlung von praktischen Sezierübungen, welche unter dem Titel „Anatomia Mundini“ zuerst im Jahr 1475 in Padua gedruckt wurde [16]. Bis zur Veröffentlichung von Vesals „De humani corporis fabrica“ war die „Anatomia Mundini“ das maßgebliche Lehrbuch auf dem Gebiet der Anatomie. Für Bologna war die Anzahl der Teilnehmer bei der Sektion einer männlichen Leiche auf 20, bei einer weiblichen auf 30 Personen festgelegt [16]. Eine anatomische Sektion dauerte 4 Tage. Am ersten Tag wurden das Abdomen präpariert, am zweiten Tag der Brustkorb, am dritten Tag der Kopf und am vierten Tag die Extremitäten mit Knochen, Muskeln und Blutgefäßen. Seit der alexandrinischen Zeit war es das erste Mal, dass Leichen wieder systematisch untersucht wurden. Anatomische Studienobjekte waren Gehenkte, Selbstmörder oder Getötete, denen nach kirchlicher Auffassung kein ehrenvolles Begräbnis zustand [3, 6, 14–17]. Eine medizinische Fakultät mit eigener rechtsverbindlicher Verfassung gab es in Bologna erst im Jahr 1405.

### Padua

Im Jahr 1250 hat der medizinische Unterricht in Padua begonnen, im Jahr 1424 gab es bereits 9 medizinische Lehrstühle [4, 14, 15, 17]; 2 für Theorie, Praxis und Chirurgie sowie 3 für Philosophie. Der Unterricht wurde in der Regel in den Morgenstunden abgehalten. Auf Verlangen der Studenten musste seitens der Professoren auch Einzelunterricht erteilt werden. Interessanterweise war es den Lehrenden verboten, den Unterrichtsstoff vorzulesen. Bei Zuwiderhandlungen des Lehrkörpers und bei Vernachlässigung des Unterrichts gab es Strafmaßnahmen. Während im 14. Jahrhundert die Studenten ihren Lehrkörper noch selbst gewählt und auch bezahlt haben, ging mit der Finanzierung der Hochschulen durch die Regierung das Privileg der studentischen Wahl der Professoren verloren. Die Herrschaft Venedigs über Padua (1405–1797) übte einen positiven Einfluss auf das dortige akademische Geschehen aus, zumal der Senat von Venedig die große Bedeutung der Universitäten richtig einzuschätzen wusste und sich auch selbst um die Wahl der besten Professoren gekümmert hat [14, 15, 17, 18]. Der Senat handelte nach dem Prinzip, der Universität größte Freiheiten und Privilegien einzuräumen. Im 15. Jahrhundert und während der gesamten Renaissance hat sich Padua zu einem der größten Kulturzentren Europas entwickelt. Die besondere Bedeutung dieses Lehrzentrums ist auch daran erkennbar, dass sich ausländische Herrscher insbesondere um Ärzte und Juristen aus Padua bemüht haben. Im Jahr 1558 wurde der klinische Unterricht erstmalig in der Form eingeführt, dass Ärzte im Franziskus-Hospital Vorträge am Krankenbett gehalten haben.

Seit 1490 hat Alessandro Benedetti (1450–1512) in Padua Anatomie und praktische Medizin gelehrt [15, 17]. Er war ein Verfechter regelmäßig durchgeführter anatomischer Sektionen. Die Sektion dauerte 5 Tage: Am ersten Tag erfolgten die Inspektion des Körpers und die Präparation von Knochen und Muskeln, am zweiten Tag die anatomische Eröffnung des Unterleibs (Urogenitaltrakt; Verdauungsapparat), am dritten Tag die Exploration von Lunge, Herz und Leber, am vierten Tag die des Gehirns und am fünften Tag die der Gefäße und Nervenbahnen. Benedetti empfahl die Einrichtung eines beständigen Gebäudes zur Durchführung von Sektionen. Das anatomische Theater sollte an einem großen und luftigen Ort erbaut werden, von der Form vergleichbar einem Amphitheater oder einer Arena. Die Leiche sollte auf einem erhöhten Tisch im Zentrum liegen, für alle Zuschauer gut sichtbar.

## Andreas Vesal und die *Humananatomie*

Erst der Belgier Andreas Vesalius (1514–1564) hat die wissenschaftlich fundierte Humananatomie begründet und auf die Fehler der Anatomie des Galen aufmerksam gemacht [2, 3, 6, 15–18]. Vesalius, der im Alter von 23 Jahren zum Professor der Chirurgie und Anatomie an die Universität Padua berufen wurde, veröffentlichte in 1. Auflage 1543 sein Hauptwerk über die Anatomie „De humani corporis fabrica libri septem“ („Sieben Bücher über den Bau des menschlichen Körpers“, Basel 1543). Zur Anfertigung der Abbildungen hatte Vesal den Tizianschüler Jan Stephan van Calcar (1499–1546) beauftragt. Viele Vorstellungen Galen’s hat Vesal widerlegt, zur Theorie der Poren in der Herzscheidewand hat er sich nicht verbindlich geäußert. Galen war der Auffassung, dass das Blut von der rechten in die linke Herzkammer durch Poren in der Herzscheidewand fließt. Trotz der neu gewonnenen Erkenntnisse haben viele Anatomen weiterhin die Schriften Galens gelehrt und Vesals Anatomie heftig angegriffen. Vor allem Vesals Lehrer, der Pariser Anatom Jacobus Sylvius (1478–1555), verfasste eine bösartige und beleidigende Schmähschrift gegen seinen Schüler [2, 6]. Er bezeichnete ihn als einen Verrückten, der mit seinem giftigen Hauch Europa verpeste. Wie unglaublich erscheint es uns heute, dass über die Richtigkeit einer Beobachtung aus der beschreibenden Anatomie so große Zweifel entstehen konnten! Vesal hat den Lehrstuhl in Padua 1544 im Alter von 30 Jahren verlassen, um Leibarzt Kaiser Karl des V. zu werden.

Das 16. Jahrhundert ist das goldene Zeitalter der Anatomie Paduas. Nachdem Vesal Padua verlassen hatte, übernahm Realdo Colombo (1516–1559) seinen Lehrstuhl. Colombo, der noch nichts vom großen Blutkreislauf wusste, hat im Jahr 1559 den pulmonalen Blutkreislauf entdeckt. Weitere berühmte Anatomen Paduas waren Gabriele Fallopio (1523–1562), Fabrizio d’Aquapendente (1537–1619) und Giovanni Battista Morgagni (1682–1771) [2, 17, 18]. Um 1594 entstand das neue Amphitheater für Anatomie. Fabrizio d’Aquapendente, der in Padua den Lehrstuhl für Chirurgie und Anatomie innehatte, war einer der herausragenden Gelehrten seiner Zeit [17]. Er verfasste zahlreiche wissenschaftliche Abhandlungen zu unterschiedlichsten Themen wie der Atmung und dem Blutstrom und gilt als Entdecker der Venenklappen. Nach dem Tod von Fabrizius fand man unter seinen Büchern eine handschriftliche Kopie des Buches „Exercitatio de motu cordis et sanguinis in animalibus“ seines englischen Schülers William Harvey (1578–1657) [17].

## Leonardo da Vinci und die Anatomie

Ein großer Impuls für die Anatomie ging auch von der bildenden Kunst aus. Die immer realistischer werdenden Darstellungen des menschlichen Körpers haben dazu beigetragen, das Interesse an anatomischen Studien zu wecken. So hat Leonardo da Vinci (1452–1519) in Florenz Sektionen an Leichen durchgeführt und eine Vielzahl anatomischer Zeichnungen von hervorragender künstlerischer Qualität und Anschaulichkeit angefertigt [19, 20]. Leonardo war kein Arzt, und seine anatomischen Studien hat er nicht veröffentlicht. Von 1570 bis spät in das 18. Jahrhundert waren seine Zeichnungen verschollen. Ein halbes Jahrhundert vor den anatomischen Zeichnungen des Vesal hat Leonardo ungeahnte Blicke in die dreidimensionale Wirklichkeit des Körperinneren ermöglicht. Zum Verständnis der Funktionen von Atmung, Herztätigkeit und Wärmebildung benutzte er mechanische und physikalische Gesetzmäßigkeiten.

## Klinischer Unterricht am Krankenbett

Padua ist auch der Ort, an dem erstmalig der klinische Unterricht am Krankenbett praktiziert wurde [14, 15, 17]. Giovanni Battista da Monte (Montanus, 1498–1551) hat in den Sälen des Spitals San Franceso Vorlesungen gehalten und die Patienten von den Studenten untersuchen lassen. Montanus hat sich von seinen Studenten ins Hospital begleiten lassen, um sie dann in der praktischen Medizin zu schulen. Die Krankengeschichte wurde erfasst und der Patient unter den Augen des Professors untersucht. Diese Form des Unterrichts muss die Studenten wohl sehr beeindruckt haben. Der Holländer Jan van Heurne (1543–1601), der auch in Padua studiert hat, hat ihn später in Leiden praktiziert [15, 17, 21, 22]. Die Einführung des praktischen Unterrichts am Krankenbett war zweifellos ein klinisch herausragendes Ereignis. Uns erscheint es heutzutage merkwürdig, warum einige der damaligen Professoren gegen die Einführung des systematischen Unterrichts am Krankenbett opponiert haben. Viele Professoren hatten sich jedoch an die Form des Bücherstudiums und den Vortrag am Katheder gewöhnt, es war ihnen lästig, am Krankenbett zu unterrichten. Außerdem erachteten sie die Lehre am Krankenbett als nicht wissenschaftlich.

Die Lehrer am Krankenbette, die Kliniker, konnten erst allmälig durch hervorragende Aerzte am Krankenbette selbst erzogen worden. (Theodor Billroth, 1829–1894, [23])

In der zweiten Hälfte des 15. Jahrhunderts herrschte in der Medizin wie in der Philosophie und auch der Literatur und Kunst der Geist der Renaissance vor. Man orientierte sich in der Medizin wieder an den klassischen griechischen und lateinischen Texten [2, 6]. Im 16. Jahrhundert führten die religiösen Machtkämpfe in Deutschland und anderen europäischen Ländern zur Reformation. Die kirchlichen Behörden sahen die protestantische Studentenbewegung skeptisch und nicht ohne Besorgnis. Die Kirche hatte bei den Hochschulen eine durchaus strenge Überwachungsfunktion, die sie nicht verlieren wollte. In der Organisation des Unterrichts der europäischen Universitäten änderte sich während des 16. und 17. Jahrhunderts nur wenig. Bei den medizinischen Fakultäten standen die Vorlesungen im Vordergrund, die praktische Demonstration hatte allenfalls marginale Bedeutung [3, 4]. Freie Vorträge wurden selten gehalten, der Lehrstoff bestand aus alexandrinischen, griechischen, römischen und arabischen Elementen. Dies hätte vorausgesetzt, dass der Lehrer sein Fach gründlich beherrschte und auch der lateinischen Sprache mächtig gewesen wäre. Der Zustand des medizinischen Unterrichts war in allen Universitäten des christlichen Abendlandes vergleichbar. Die Lehrer konnten vergleichsweise formlos und freizügig unterrichten.

## Die Entdeckung des Herz-Kreislauf-Systems – die Zirkulation

Seit 1616 ist durch den Engländer William Harvey (1578–1657) bekannt, dass Blut beständig in unserem Körper zirkuliert. In Padua hat Harvey bei seinem Lehrmeister Fabrizio d’Aquapendente von der Entdeckung der Venenklappen und den Experimenten zum Blutfluss gehört. Jene Venenklappen werden in Harveys Theorie zu einem wichtigen Bestandteil der Erklärung des Kreislaufs. Harvey erkennt und beweist als Erster, dass das Blut im Körper einem Kreislauf folgt. Vom Herzen wird es über Arterien in den Körper geleitet, durch die Venen strömt es zurück zum Herzen [20, 24, 25]. In der Vorrede seines Werkes „Exercitatio anatomica de motu cordis et sanguinis in animalibus“ („Anatomische Studie über die Bewegung des Herzens und des Blutes in den Tieren“), erschienen 1628 in Frankfurt/Main, geht er bei der Widerlegung der Thesen von Galen auch auf dessen Atmungslehre ein. Die Schrift gilt als erste grundlegende experimentelle Arbeit der Physiologie, in welcher Hypothesen durch Fakten, Experimente und mathematische Berechnungen verifiziert werden konnten. Harvey würdigt die Klassiker Aristoteles und Galen, gleichzeitig aber auch die großen italienischen Anatomen. Von den vielfältigen Entdeckungen der Anatomie sollen noch die von dem italienischen Anatomen Giovanni Battista Morgagni erarbeitete sog. „Solidarpathologie“ sowie die von dem Schweizer Albrecht von Haller begründete „Sensibilitäts- und Irritationstheorie“ erwähnt werden [2, 6, 22, 26]. Morgagnis Werk „De sedibus, et causis morborum per anatomen indagatis libri quinque“ („Fünf Bücher über die Lokalisation und die Ursachen der Krankheiten mittels anatomischer Erforschung“; Venedig 1761) stellt die endgültige Überwindung der Theorien von Galen und seinen Vorgängern („Humoralpathologie“) dar. Hallers Vivisektionsexperimente an Tieren haben die Grundlagen zur Beschreibung der „Sensibilitäts- und Irritationstheorie“ gelegt.

## Leiden als Keimzelle des klinischen Unterrichts in Europa und der Welt

Im Zeitraum vom 17. bis 19. Jahrhundert wurde das mittelalterlich geprägte religiöse Weltbild in Europa fundamental infrage gestellt. Die Vorstellung, dass die Natur und das Leben ausschließlich durch geheime Mächte und die Kirche geprägt seien, wurde abgelegt. Mit der Etablierung der Naturwissenschaften ergab sich plötzlich eine Berechenbarkeit. Man brauchte nicht mehr zu magischen Mitteln zu greifen, um Schamanen, Götter und böse Geister zu besänftigen. Der Blick in das Innere des menschlichen Körpers hat sich versachlicht und gegenüber früher an Unheimlichkeit verloren. Das 17. Jahrhundert wurde zu einer Blütezeit experimenteller anatomischer Forschung. Es entstanden grundlegende Arbeiten über das Herz-Kreislauf-System, die Lunge sowie die Lymphwege und die Drüsen [2, 3, 6].

Im 17. und 18. Jahrhundert waren die niederländischen Universitäten führend, wobei insbesondere die Universität in Leiden alle übertraf [16, 17, 27–31]. Leiden hatte zu damaliger Zeit brillante Kliniker und medizinische Lehrer [21, 22]. Zuerst war es Otto van Heurne (1577–1652), der als Student in Padua gewesen ist, dann Franciscus Sylvius (Franz de le Boe; 1614–1672) und später v. a. Hermann Boerhaave (1668–1738). Sylvius hat als Verantwortlicher für die praktische Medizin den Unterricht am Krankenbett als Teil des medizinischen Curriculums etabliert. Sein Enthusiasmus in der studentischen Lehre war so groß, dass er die zuvor nur an 2 Tagen der Woche durchgeführten Visiten am Krankenbett täglich praktiziert hat [21, 28]. Nach seiner Theorie basierte medizinisches Lernen auf 3 Säulen: dem Unterricht am Krankenbett, der anatomischen Betrachtung sowie dem Experiment. Im Spital „St. Caecilie Gasthuis“ hat Sylvius praktische Übungen und Unterricht am Krankenbett, ganz nach dem Vorbild der Schule von Padua, durchgeführt. Dazu standen ihm Patienten aus 2 Abteilungen mit je 6 Betten für Männer und Frauen zur Verfügung.

Den Professoren in Leiden gebührt das besondere Verdienst, den Unterricht am Krankenbett als feste Institution des medizinischen Studiums etabliert zu haben. Unter Leitung von Sylvius und seinem Nachfolger Boerhaave genoss die Leidener Universität einen solch hervorragenden Ruf, dass Studenten und Ärzte aus aller Welt nach Leiden kamen, um dort in die medizinische Lehre zu gehen [21, 22]. Boerhaaves Unterricht war nicht dogmatisch geprägt. Im Vergleich zur „trockenen“ Theorie der Bücher vermittelte er Theorie und praktische klinische Medizin in Verbindung mit persönlicher Einschätzung, Bewertung und anekdotischer Darstellung. Nicht die aus dem Buch gelernte Theorie war Ziel der medizinischen Ausbildung, sondern die bewusste Wahrnehmung des eigentlichen Subjekts, die des kranken Menschen. Boerhaave hat den Unterricht am Krankenbett nicht erfunden! Er hat jedoch verstanden, dass die praktische Medizin elementar auf die Wahrnehmung und Verlaufsbeobachtung von Zeichen und Symptomen am Kranken angewiesen ist. Die von den Schülern Boerhaaves aufrichtig vermittelte Verehrung und Dankbarkeit hat sich in Bezeichnungen wie „unser Professor“, „der große Boerhaave“, „the Dutch Hippokrates“ oder der „Communis Europae Praeceptor“ widergespiegelt [21]. Boerhaave ist so bekannt gewesen, dass selbst von fernen Ländern abgesandte Post mit dem Titel „To the Greatest Physician in the World“ den Weg nach Leiden gefunden hat. Boerhaave hat der Universität Leiden einen Glanz verliehen, der sich bis weit ins 18. Jahrhundert erstreckt hat.

Die beiden berühmtesten Schüler Boerhaaves waren zweifellos Gerhard van Swieten (1700–1772) und Albrecht von Haller (1708–1777). Zusammen mit Anton van Haen (1704–1766), ebenfalls ein Boerhaave-Schüler, wurde van Swieten Begründer der sog. „Ersten Wiener Medizinischen Schule“, die Wien fortan zum medizinischen Zentrum von Europa gemacht hat [31]. Der Schweizer Albrecht von Haller, der zusammen mit Boerhaave den Grundstein für die moderne Physiologie gelegt hat, bekam 1736 in Göttingen den Lehrstuhl für Anatomie, Chirurgie und Botanik. Alexander Monroe (1697–1762), ebenfalls ein Schüler von Boerhaave, ist 1720 zum Professor der Anatomie an der neu gegründeten Universität Edinburgh berufen worden. Viele Schüler Boerhaaves haben das Wissen ihres Lehrmeisters in die Welt hinausgetragen, nicht nur an den Ort ihres zukünftigen Wirkens, sondern auch in ferne Länder durch Übersetzung zahlreicher Schriften und Kommentare Boerhaaves [21, 31]. Es hat später kaum eine medizinische Fakultät in Europa gegeben, an der nicht ein Boerhaave-Schüler praktiziert und/oder gelehrt hat.

## Zusammenfassung

Das Wissen vom Aufbau des Körpers sowie den Funktionen der Organsysteme bleibt die Basis für jedes ärztliche Handeln. Eine grundlegende anatomische Ausbildung unserer Medizinstudenten ist deshalb weiterhin elementar und ohne die Möglichkeit der eigenen Anschauung kaum vorstellbar. Sowohl die traditionellen als auch die modernen Methoden medizinischer Diagnostik und Therapie erfordern einen umfassenden anatomischen Wissensstand. Anhand dieses geschichtlichen Rückblicks soll neben der Würdigung anatomisch-naturwissenschaftlicher Entwicklung und Lehre aber auch an die notwendige Hinterfragung von Autoritäten und Dogmen erinnert werden. Insbesondere in der Medizin darf Wissen nicht als Dogma verstanden und in diesem Sinne gelehrt werden. Die Geschichte zeigt eindrucksvoll, dass neues Wissen und vermeintlich „Richtiges“ beständig hinterfragt werden müssen. Und noch eines: Es wäre ein großer Fehler, sich bei allem wissenschaftlichen Fortschritt nicht mehr auf die medizinhistorische Betrachtung zu besinnen. Deshalb schmerzt es auch, wenn Studenten am Ende ihrer medizinischen Ausbildung nicht einmal wissen, wer Galen, Vesal oder Harvey waren.
